# The relations between executive functions and occupational functioning in individuals with bipolar disorder: a scoping review

**DOI:** 10.1186/s40345-022-00255-7

**Published:** 2022-03-14

**Authors:** Juul Koene, Susan Zyto, Jaap van der Stel, Natasja van Lang, Marion Ammeraal, Ralph W. Kupka, Jaap van Weeghel

**Affiliations:** 1 University of Applied Sciences Leiden, Leiden, The Netherlands; 2 Tranzo, Scientific Centre for Care and Wellbeing, Tilburg University, Tilburg, The Netherlands; 3 Department of Psychiatry, Amsterdam University Medical Center, Vrije Universiteit, Amsterdam, The Netherlands; 4 Mental Health Service Organisation North Holland North, Hoorn, The Netherlands; 5 GGZinGeest Center for Mental Health Care, Amsterdam, The Netherlands; 6 Phrenos Center of Expertise, Utrecht, The Netherlands

**Keywords:** Executive functions, Occupational functioning, Self-regulation, Emotion regulation, Bipolar disorder, Scoping review

## Abstract

**Background:**

Patients with bipolar disorder experience impairments in their occupational functioning, despite remission of symptoms. Previous research has shown that neurocognitive deficits, especially deficits in executive functions, may persist during euthymia and are associated with diminished occupational functioning.

**Objectives:**

The aim of this scoping review was to identify published studies that report on the relationships between executive functions and occupational functioning in BD to review current knowledge and identify knowledge gaps. In addition to traditional neuropsychological approaches, we aimed to describe executive functioning from a self-regulation perspective, including emotion regulation.

**Methods:**

We applied the methodological framework as described by Arksey and O’Malley (Int J Soc Res Methodol Theory Pract 8:19–32, 2005) and Levac et al. (Implement Sci 5:1–9, 2010). We searched PubMed and psycINFO for literature up to November 2021, after which we screened papers based on inclusion criteria. Two reviewers independently performed the screening process, data charting process, and synthesis of results.

**Results:**

The search yielded 1202 references after deduplication, of which 222 remained after initial screening. The screening and inclusion process yielded 82 eligible papers in which relationships between executive functions and occupational functioning are examined.

**Conclusion:**

Neurocognitive deficits, including in executive functions and self-regulation, are associated with and predictive of diminished occupational functioning. Definitions and measurements for neurocognitive functions and occupational functioning differ greatly between studies, which complicates comparisons. Studies on functional remediation show promising results for improving occupational functioning in patients with BD. In research and clinical practice more attention is needed towards the quality of work functioning and the various contexts in which patients with BD experience deficits.

**Supplementary Information:**

The online version contains supplementary material available at 10.1186/s40345-022-00255-7.

## Introduction

Bipolar disorders (BD) are characterized by episodes of depression and (hypo)mania, alternating with periods in which patients experience a remission of symptoms, euthymia (Apa 2013). Although originally viewed as episodic with full recovery during euthymia, it has become evident that many euthymic patients despite achieving clinical recovery still experience functional impairments in various domains of daily life (Tohen et al. 2003; Wingo et al. 2010). These impairments are especially present in the area of employment and occupational functioning, as shown by high unemployment numbers and reported difficulties at work (Gilbert and Marwaha 2013). Individuals with BD experience problems with finding and maintaining stable employment matching their capabilities. Unemployment rates range from 40 to 60%, a percentage showing little variation across European countries (Morselli et al. 2004; Huxley and Baldessarini 2007). Furthermore, employed individuals with BD report considerable difficulties with job retention and frequent problems at work such as underemployment (Marwaha et al. 2013), absenteeism (Dean et al. 2004), and reduced productivity (McMorris et al. 2010).

Occupational functioning can be categorized into work participation and work functioning (Sandqvist and Henriksson 2004; Lagerveld et al. 2010). *Work participation* is the extent to which the individual participates in the job market. *Work functioning* refers to skills, attitudes, behavior, and work performance of the individual, and relates to the output (e.g. performance) in relation to the input (productivity, behavior, skills). Adequate work functioning is a prerequisite for successful work participation. Individuals with BD experience problems in both work participation and functioning. As such, impaired occupational functioning is a relevant target for treatment and rehabilitation in BD care, and in recent years several studies have focused on the experienced problems and associated factors (Miskowiak et al. 2017).

Various factors impact occupational functioning of patients with BD, including the overall severity of the disorder and subthreshold depressive symptoms (Burdick et al. 2010a; Rosa et al. 2009), next to deficits in neurocognitive functions (Depp and Mausbach 2012; Martinez-Aran et al. 2007). It has been hypothesized that an underlying neurobiological process of illness progression (also conceptualized as neuroprogression) may be the cause of increasing functional impairment in at least a subgroup of patients with BD (Neuroprogression 2009). Research has shown that neurocognitive impairments persist across mood states and are linked to various problems in everyday functioning, including employment (Martinez-Aran et al. 2007; Depp et al. 2012b). Especially impairments in verbal memory and executive functions (EF) have been implicated as important factors for occupational recovery (Tse et al. 2014; Ferrier et al. 1999; Mur et al. 2009). Together with residual depressive symptoms, level of education, and cognitive deficits, particularly in EF and verbal memory, are among the best predictors of employment in BD (Gilbert and Marwaha 2013).

The association between EF deficits and occupational functioning in patients with BD has led to a growing scientific interest, resulting in various empirical studies, literature reviews, and meta-analyses (Depp et al. 2012b; Tse et al. 2014; Baune and Malhi 2015). EF are generally described as higher cognitive processes that regulate lower level processes, and are viewed as core mechanisms for the overarching ability of self-regulation (Snyder et al. 2015; Nigg 2017). Self-regulation refers to the ability to regulate internal processes (cognition, emotion, and behavior) to adapt to novel situations, manage goal-directed actions, and co-operate with others (Nigg 2017; Barkley 2012). The aspect of self-regulation concerned with emotion regulation is specifically of interest, as both mood and emotion are affective states whose regulation can be problematic for patients with bipolar disorder (Larsen 2000; Gross 2014). Furthermore, deficits in EF may be elevated in situations with emotional stimuli, and EF are considered engaged when regulating emotions (Lima et al. 2017). Both EF and self-regulation are associated with successful functioning in daily life, including occupational functioning (Diamond 2013; Moffitt et al. 2011). As such, both EF and self-regulation are promising targets for research and treatment in relation to occupational functioning (Cramm et al. 2013).

In this review, we have the following research question: “What is known about the relationship between deficits in executive functioning and self-regulation, and occupational functioning in patients with bipolar disorders?” For this purpose, we aimed to (1) summarize the studies that investigated this relationship; (2) clarify the definitions and measurements used for executive functioning, self-regulation, and occupational functioning in these studies; and (3) give a description of how the relationship is measured and what is known about the strength of this relationship.

## Method

Scoping reviews are a specific form of literature review, in which broad research questions are addressed and an overview of literature can be presented. This type of literature review is suited to map research within a particular field and clarify working definitions. Scoping reviews differ from systematic reviews because authors typically do not assess the quality of included literature (Levac et al. 2010) and are conducted to clarify definitions and identify gaps in literature. Considering our broad and conceptual question, we deemed a scoping review to be the most applicable methodology. As such, we used the six stages as proposed by Arksey and O'Malley (2005): (1) identifying the research question, (2) identifying relevant studies, (3) study selection, (4) charting the data, and (5) collating, summarizing, and reporting the results. As part of the sixth and optional stage of the scoping review, (6) we consulted with an occupational therapist (MA) to discuss how the results of our study may enhance occupational therapy in patients with bipolar disorders. The PRISMA extension for scoping reviews (PRISMA-ScR) was used to draft the manuscript of the scoping review (Tricco et al. 2018).

### Search strategy

To identify relevant studies, PsycINFO and PubMed were used. There was no limitation on year of publication. An updated search was conducted with a time range from February 2018 to November 2021, after which stages 3 (study selection, screening) and 4 (charting the data) were conducted for a second time. The searches were conducted using combinations of the following search terms: (executive function* OR self-regulation OR cogniti* OR neurocogniti* OR executive OR emotion regulation OR cognitive function*) AND (vocation* OR vocational function* OR occupation* OR employ* OR work OR labor) AND (bipolar disorder). The strategy was discussed within the scoping study team and further refined after discussion. Language restrictions were set to English.

### Study selection: inclusion and exclusion criteria

Papers were included in the scoping review if they:

1. Described a relationship between executive functions or self-regulation and occupational functioning in adult patients (≥ 18 years) with bipolar disorder;
2. Were written in English
3. Were peer reviewed (published in a peer reviewed journal or reviewed by a dissertation committee);
4. Consisted of a quantitative and/or qualitative design.

Secondary research (i.e., reviews and meta-analyses) and dissertations were also included to investigate the breadth of the subject. Papers were excluded if the studies focused on medication, pediatric bipolar disorder/participants younger than 18 years, or if the paper did not consist of original or secondary research (e.g., study protocols).

### Screening procedure

#### Initial screening

An initial screening based on the inclusion and exclusion criteria was performed by JK or SZ. In case of doubt, the paper was transferred to next phase (title and abstract screening). Most excluded references focused on medication or on populations beyond the scope of this review (e.g., a sole focus on schizophrenia).

#### Title and abstract screening

A relevance tool based on the inclusion an exclusion criteria was developed Additional file 1: Appendix S1. References were imported into an Excel sheet and were divided randomly among the co-authors (JvW, RK, JvdS, NvL). Each reviewer received a randomized sample from the potentially relevant articles. JK or SZ functioned as second reviewer for each of the reviewers.

After independently screening the titles and abstracts, the reviewers met to discuss their findings and resolve discrepancies. Discrepancies in scoring for relevance were mostly due to the inclusion criterium “EF or self-regulation in the context of employment”, i.e., in some titles and abstracts of the selected articles, this inclusion criterium could not be scored. If this was the case, the article was transferred to the next phase.

#### Full paper screening

For the purpose of screening the full papers, the relevance tool was further specified by the reviewers of the scoping study team, because some criteria needed more refinement to enhance the reviewing process (see Additional file 1: Appendix S1 and Additional file 2: Appendix S2). The main point was a diagnosis check in order to be eligible for inclusion. For the full paper screening a similar procedure was used as in the title and abstract screening: each reviewer received a random sample of the full papers. JK, or SZ, reviewed the papers as second reviewers.

### Data charting process

A data charting form (see Additional file 3: Appendix S3) was developed to extract relevant information and study characteristics (e.g., design, study population, measurements). JK and NvL pilot tested the form for 10 randomly chosen references. Discrepancies were discussed until consensus was reached. After the pilot test, the data charting form was revised and a separate form for secondary research (e.g. literature reviews) was developed (see Additional file 4: Appendix S4). The final version of the data charting form was used throughout the rest of the data charting process. This final form captured key concepts: executive functioning, occupational functioning, clinical characteristics of the participants, and the relationship between executive functions and occupational functioning.

**Fig. 1:**
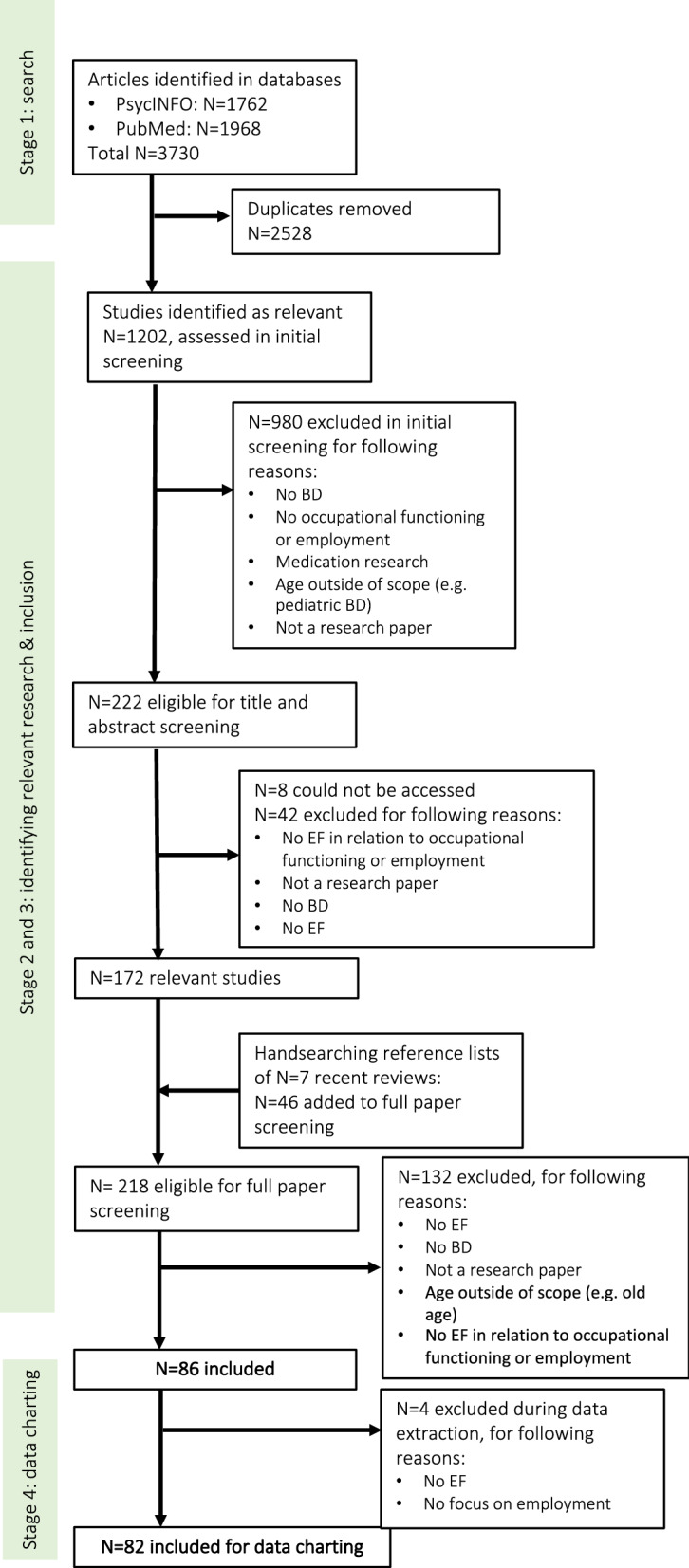
PRISMA flow chart of the study selection process

SZ, NvL, JvW, RK, and JvdS participated as reviewers in the data charting process. JK or SZ acted again as the second reviewer. After independently filling out the forms, the reviewers met to discuss the results. It appeared that there were no disagreements between the reviewers, only some inconsistencies. These inconsistencies were easily resolved, since they were related to misinterpreting text on for instance diagnosis check, description of episodes, or definitions of executive and vocational functioning. The results of this screening process are shown in Fig. 1 (flow chart).

### Analysis of results

Information from the data charting forms was collated in a Microsoft Excel spreadsheet for further analysis and thematic organization. We initially focused on numerical data about study characteristics, such as research design, study population, and course of illness. This provided an overview of the nature of the included studies. Secondly, we collated data from the data charting forms and Excel spreadsheet to organize the information in themes relevant to the second aim of this scoping review, i.e., clarifying definitions and measurements of the neurocognitive deficits and occupational functioning used. We categorized the included papers in work *participation* and work *functioning* based on the description as stated in the introduction. Work *participation* was measured in a dichotomous yes/no manner or by further categorization of work participation (e.g., full-time, part-time, retired, disability leave). Work *functioning* was measured by the quality of the described functioning. Neurocognitive deficits and specifically EF and self-regulation were described by how they were defined and measured in the included studies. With regard to the third aim of this review, the manner in which the relationship between the neurocognitive deficits and occupational functioning was investigated, was summarized, as well as information about the strength of this relationship. For this aim, we collected the effect size (ES) from the studies, if present. If we couldn’t extract the ES we calculated them when enough information was present. If the ES were expressed as r, ‘d, odds ratios, R^2^ or η^2^ they could be used. β-Coefficients however were calculated into *f*^2^. The ES are presented qualitatively (i.e. small, medium, large) together with the direction of the association.

## Results

**Fig. 2.**
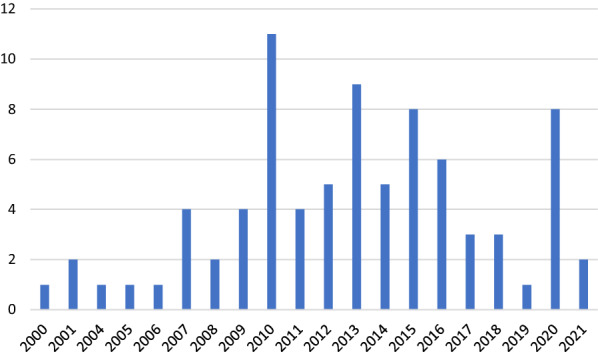
Published studies per year

The search strategy yielded, after deduplication, 1202 references. Based on the initial screening, N = 222 references remained and were included for the title and abstract screening. Most excluded references focused on medication or on populations beyond the scope of this review (e.g., a sole focus on schizophrenia). From the title and abstract screening phase, 172 articles were deemed relevant to include for the full paper screening phase. Of these 172 references, 155 documents were procured by using database subscriptions and 9 documents were procured via VU Medical Centre librarians and contacting corresponding authors. Eight papers could not be accessed. Of these 172 papers, two were excluded at this point due to lacking relevance. Additionally, bibliographies of reviews published in the last 5 years (starting in 2013; 7 reviews) were hand-searched to check for any relevant literature missed in the electronic search. After deduplication, 46 potentially relevant papers were added to the full paper screening. In total, 218 full papers were screened for relevance. From the full paper screening phase, a total of 69 articles were initially included for the data charting process. The additional search was conducted in the same manner, and yielded 13 additional references for inclusion in the data charting process. Papers were mostly excluded because they did not examine the possible relationship between EF and occupational functioning. In total 82 papers remained after the full paper screening and were included in this review (see Additional file 5: Appendix S5). The included papers consisted of original research (67 of 82 included papers: 44 cross-sectional studies, four intervention studies, 19 longitudinal studies), and secondary literature such as literature reviews and meta-analyses (14 of 82 papers: two meta-analyses, eight (qualitative) reviews, four systematic reviews). Included studies were published between the years 2000–2021, with most studies (11) published in the year 2010, nine studies published in 2013, and eight in 2015 (Fig. 2). Most studies (30) originated from the United States of America, followed by Spain (17), and Australia (eight). Table 1 shows the geographic distribution of published studies.

**Table 1:** Studies by country of publication

Country	Number
USA	30
Spain	17
Australia	8
Denmark	4
UK	3
New Zealand	2
China	2
Canada	2
Turkey	1
Czech Republic	1
Brazil	1
France	1
Norway	1
The Netherlands	1
Hong Kong	1
Mexico	1
Greece	1
Sweden	1
Argentina	1
Germany	1
Taiwan	1
Nigeria	1

### Definitions of concepts

#### Definitions of occupational functioning

Occupational functioning was defined in 23 of the 82 included papers (Wingo et al. 2010; Gilbert and Marwaha 2013; Martinez-Aran et al. 2007; Depp et al. 2012b; Tse et al. 2014; Lomastro et al. 2020; Altshuler et al. 2007; Bello 2009; Bonnin et al. 2014; Bowie et al. 2010; Deckersbach et al. 2010; Drakopoulos et al. 2020; Duarte et al. 2016; Harvey et al. 2010; Levy et al. 2013; Luo et al. 2020; Mur et al. 2008; Sanchez-Moreno et al. 2009; Solé et al. 2018; Strassnig et al. 2017; Tabarés-Seisdedos et al. 2008; Torrent et al. 2007; Wilder-Willis 2001). We have categorized the definitions regarding the two aspects of occupational functioning mentioned in the introduction: work participation and work functioning. From the remaining 58 papers that did not give a definition of occupational functioning, 32 studies reported on work participation, and 15 studies reported on work functioning without explicitly defining this. Eleven studies included a description that could be attributed to both work functioning and participation. In one paper, a review by Boland and Alloy (2013), there was no report on occupational status. This review does report, however, on the impact of cognitive variables on employment trajectory and the relation with sleep.

Given definitions most often entail *work participation* and were mostly based on the dichotomy of employment versus unemployment (Gilbert and Marwaha 2013; Martinez-Aran et al. 2007). In some studies, the extent to which an individual participated in the job market was further clarified by categorizing employment. This could be described as “part- or full-time employment in compensated, non-supportive settings” (Strassnig et al. 2017), by giving a percentage (working or studying more or less than 50% (Drakopoulos et al. 2020) or specifying the hours per week the individual is working (Altshuler et al. 2007; Lewandowski et al. 2020). Unemployment was most basically defined as “not working”, “not being not able to work”, being “retired/disabled” (Mur et al. 2008) or “inactive” (Drakopoulos et al. 2020).

Other definitions of work participation are embedded in a broader context of social and occupational functioning in general, as the “ability to perform activities of daily living such as handling household, working or studying” (Solé et al. 2018), “the capacity or performance on daily tasks that are essential for maintenance of social and occupational roles” (Depp et al. 2012b) or “the capacity to fulfill role requirements as a worker or student”, next to other social roles (Wilder-Willis 2001). Harvey et al. (2010) describe occupational functioning in the context of functional recovery in which functional recovery refers to “regaining highest levels of premorbid occupational and residential status” further imbedded in “the ability to perform tasks relevant to everyday”.

Regarding *work functioning*, which we have described as the skills, behavior, and attitudes needed for carrying out work, eight papers have given a definition of occupational functioning that is (partly) comparable with this description of work functioning (Martinez-Aran et al. 2007; Depp et al. 2012b; Bowie et al. 2010; Deckersbach et al. 2010; Duarte et al. 2016; Harvey et al. 2010; Sanchez-Moreno et al. 2009; Solé et al. 2018). The definitions provided in these studies differ from each other, but most describe the capacities essential for carrying out work or fulfilling occupational roles (Martinez-Aran et al. 2007; Depp et al. 2012b; Harvey et al. 2010). The description of work functioning is sometimes embedded in a definition of psychosocial functioning (Solé et al. 2018; Lewandowski et al. 2020) or referred to in the context of work impairment, which is “the impact of illness on a person’s ability to work, impairment in occupational role performance and reduced work productivity associated with output, in relation to input” (Sanchez-Moreno et al. 2009).

Other definitions of work functioning involve counting missed productivity at work, categorized in “absenteeism”, which is defined as the number of missed days of work, and “presenteeism”, which is a measure of productivity at work, translated into an equivalent of lost workdays (Deckersbach et al. 2010). Bowie et al. (2010) point out that with the use of objective performance-based measures, real differences in functioning might better be revealed than with the dichotomous or categorical definition. Bowie’s definition of occupational functioning therefore comprises work skills such as the “level of supervision needed to complete a task”, “punctuality” and “ability to stay on task and complete tasks”.

Some studies refer to occupational functioning as adaptive functioning or adjustment. It depends on the study whether this description can be characterized as either work participation or work functioning. Some studies further categorize the concept into good or bad adaptive functioning, which may correspond with “unemployment”, “partially employed”, and “employed” (Levy et al. 2013), which can be considered as work participation. Other studies defined occupational functioning based on “good” or “low occupational adaptation” (Martinez-Aran et al. 2007; Tabarés-Seisdedos et al. 2008; Torrent et al. 2007) referring to the level of functioning as being “acceptable” (without any further elaboration) or with “moderate to severe difficulties” in the job functioning. Bonnín et al. (2014) defined occupational functioning as “good” or “poor work adjustment”. Good adjustment translates into part-time or full-time competitive employment, with a more elaborate definition of bad adjustment when receiving disability payment, experiencing job instability/repeated loss of work or sheltered or non-competitive employment. Lomastro et al. (2020) defined high functioning BD as at least being able to work 30 h per week in gainful employment, as a homemaker, or as a full-time student.

#### Definitions of executive functions

Definitions of EF were given in 11 of 82 included the papers (Deckersbach et al. 2010; Boland and Alloy 2013; Depp et al. 2012a; Fulford 2011; Jiménez et al. 2012; Martinez-Camarillo et al. 2019; Miguélez-Pan et al. 2014; Varo et al. 2017; Robertson 2006; Crowe et al. 2020; Demant et al. 2015a). These definitions comprised mostly traditional views of ‘core’ EF such as inhibition, interference control, planning and cognitive flexibility (see Diamond et al. 2013 for an overview), and three papers gave definitions involving motivation, emotional intelligence, and self-regulation. None of the included papers referred to any underlying theoretical model.

EF were defined broadly as “thinking and organizing” (Crowe et al. 2020), “planning and problem-solving” (Deckersbach et al. 2010) and “processes including planning, motivation, and inhibition” (Boland and Alloy 2013) or more elaborate as “a broad range of mental abilities to respond adaptively to novel situations and are necessary for appropriate, socially responsible and effectively self-serving adult behavior” (Miguélez-Pan et al. 2014). Depp et al. (2012b) divide EF in “executive control skills” versus “reasoning/problem-solving skills”. The first skills refer to “cognitive flexibility, suppression of automatic responses and conscious allocation of cognitive resources”, whereas the latter point to “the ability to discern underlying relationships”. Fulford (2011) defines EF as “a specific set of abilities associated with planning, initiating, and monitoring complex goal-directed behavior” that are located in the frontal lobes.

One of the papers gave a definition specifically of impulsivity which was described as a “tendency to respond to stimuli without reflection or full assessment, resulting in inability to conform behavior to contextual demands” (Jiménez et al. 2012). One paper (Robertson 2006) described self-regulation, which was referred to as “the evolution of analysis, reconstruction, integration, and application of adaptive strategies” within a definition of “transformative self-regulation”. Since motivational processes are integral to definitions of self-regulation, we included a paper with a definition of work motivation. This was defined as “the psychological processes that determine the direction, intensity and persistence of action within the work” (Martinez-Camarillo et al. 2019).

### Measurements for executive functions and occupational functioning

Regarding both the measurement of EF and occupational functioning, the results show a great diversity in the instruments that are used in the included studies. Below is described which tests and measurements have been included in the studies and whether a rationale was given for this choice.

#### Measuring occupational functioning

The original research (67 of the 82 included papers) included in this review employed various measurements for occupational functioning, which are summarized in Table 2. The measurements in this table are categorized based on whether the instrument measures aspects of work participation, work functioning, or both. In 29 of the cases a reason for including the measurement was provided. Described rationales included good psychometric properties and widely used measurements in psychiatric populations.

**Table 2:** Measurements for occupational functioning

Measurement	Used in	Work participation (WP)/work functioning (WF)	Description categorization
Own questionnaire/interview/medical information	Altshuler et al. (2007), Boland et al. (2015), Bonnin et al. (2014), Crowe et al. (2020), DeTore et al. (2018), Lee et al. (2015), Levy et al. (2013), Martinez-Aran et al. (2007), Robertson (2006), Strassnig et al. (2017)	N/A	N/A
Other (e.g. categorization)	Dickerson et al. (2004), Drakopoulos et al. (2020), Duarte et al. (2016), Forcada et al. (2015), Fulford (2011), Gilbert et al. (2010), Martinez-Camarillo et al. (2019), Mora et al. (2013), Mur et al. (2008), Tabares-Seisdedos et al. (2008), Torrent et al. (2007)	N/A	N/A
Functioning Assessment Short Test (FAST)	Anaya et al. (2016), Bonnin et al. (2010), Demant et al. (2015b), Forcada et al. (2015), Jensen et al. (2015), Jiminez et al. (2012; one subscale), Liu et al. (2021), Mora et al. (2013), Rosa et al. (2013), Samalin et al. (2016), Sanchez-Autet et al. (2018), Solé et al. (2018; one subscale), Torrent et al. (2013), Varo et al. (2017), Zyto et al. (2016)	WF	Scale for occupational functioning consists of questions regarding difficulty with functioning. Such as: working in the field in which someone was educated; managing work load
Global Assessment of Functioning (GAF)	Bonnín et al. (2010), Drakopoulos et al. (2020), Forcada et al. (2015), (Gilbert et al. (2010), Kaya et al. (2007), Leany (2011), Levy et al. (2013), Luo et al. (2020), Martinez-Aran et al. (2007), Miguelez-Pan et al. (2014), Mora et al. (2013), Mur et al. (2008, 2009), O'Shea et al. (2010), Ryan et al. (2013), Van Rheenen and Rossell (2014), Sanchez-Autet et al. (2018), Simonsen et al. (2010), Tabares-Seisdedos et al. (2008)	WF	GAF assesses functioning while taking symptoms into account
Social and Occupational Functioning Assessment Scale (SOFAS)	Baune et al. (2013), Baune and Malhi (2015), Crouse et al. (2020), Duarte et al. (2016), Esan et al. (2020), Lee et al. (2013), O'Shea et al. (2010), Olley et al. (2005), Sole et al. (2012), Torrent et al. (2007), Zubieta et al. (2001)	WF	Described the degree of functioning and disability, regardless of experienced symptoms
WHO-DAS 2.0	Baune et al. (2013), Baune and Malhi (2015), Chen et al. (2021), Lee et al. (2013, 2015), Mur et al. (2009), Tabares-Seisdedos et al. (2008)	WF	There is one domain that focuses on work and the difficulties that someone may experience. These relate to finishing work, earning less money, and working at a lower level due to health problems
Social Adjustment Scale (SAS)	Baune et al. (2013), Dickerson et al. (2010), Vierck and Joyce (2015)	WF	Role functioning and performance is scored
UCSD Performance-Based Skills Assessment (UPSA)	Leany (2011), Bello (2009), Bowie et al. (2010), Depp et al. (2012b), Duarte et al. (2016)	WF	The role-play reflects general abilities needed in daily life
Social Skills Performance Assessment (SSPA)	Bowie et al. (2010)	WF	Observation of everyday tasks and social skills
Longitudinal Interval Follow-up Evaluation [LIFE(-RIFT)]	Godard et al. (2011), Wilder-Willis (2001)	WF	Describes the degree of impairment in functioning (e.g. work)
Motivation for Work Questionnaire (MWQ)	Martinez-Camarillo et al. (2019)	WF	Spanish instrument to evaluate work motivation for participating in work activities
QoL-BD	Van Rheenen and Rossell (2014)	WF	Questions regarding the quality of work functioning are present (e.g. “met demands at work”)
Social Support and Undermining Scale	Ryan et al. (2013)	WF	This questionnaire is used to examine dynamics in stressful situations, such as unemployment
Specific Level of Functoning Scale (SLOF)	Bowie et al. (2010)	WF	Questions regarding functioning, such as whether the client has employable skills, or works with minimal supervision
Structured Clinical Interview for DSM-IV (SCID)	Altshuler et al. (2007)	WP	Questions regarding the extent of work participation
PRESCA-2 (proxy for cognitive reserve)	Anaya et al. (2016)	WP	This scale categorizes occupations
Strauss-Carpenter	Baune et al. (2013), Baune and Malhi (2015), Burdick et al. (2010a)	WP	The work item consists of categorizing the rate of employment: e.g. employed continuously; employed less than half of the time in the last year
Wisconsin Quality of Life Index (WQLI)	Leany (2011), Bello (2009)	WP	Questions in the WQLI are regarding what the main activity is, what the patient would like to have as the main activity, and a question regarding satisfaction
Work and Social Adjustment Scale (WSAS)	Demant et al. (2015a, b), Jensen et al. (2015, 2016)	WP	WSAS assesses the degree of impairment in relation to work participation
WHOQoL(-Bref)	Demant et al. (2015b), Fulford (2011), Jensen et al. (2016), Lee et al. (2013, 2015)	WP	The questionnaire assesses work capacity as part of occupational functioning
Diagnostic Interview for Genetics Studies (DIGS)	Depp et al. (2012b), Ryan et al. (2013)	WP	The DIGS ascertains information on the participant’s current job and classifies it according to occupational categories (e.g., professional specialty, administrative support, unem- ployed, disabled, student)
Modified Vocational Status Index (MVSI)	Dickerson et al. (2010)	WP	The MVSI assesses categorizes occupational participation based on seven levels, ranging from full-time gainful employment to unemployed
Sheehan Disability Scale (SDS)	Drakopoulos et al. (2020), Sachs et al. (2020)	WP	Short questionnaire regarding impact of health problems. The questionnaire asks about days missed, days lost
Nam–Powers–Boyd	Fulford (2011)	WP	Categorizes occupations
Social Functioning Scale (SFS)	Kaya et al. (2007), Miguelez-Pan et al. (2014), Simonsen et al. (2010)	WP	Records occupational status, hours etc.
Vocational Status Index (VSI)	Wingo et al. (2010; minor modifications)	WP	Provides vocational status
Life Functioning Questionnaire (LFQ)	Bearden et al. (2011), Leany (2011), Bello (2009), Duarte et al. (2016), O'Donnell (2016), O'Donnell et al. (2017), Olley et al. (2005)	WP and WF	Part 1: role functioning and degree of difficulties. Part 2: degree of work participation
Multidimensional Scale of Independent Functioning (MSIF)	Baune and Malhi (2015)	WP and WF	Role functioning: e.g. full-time or part-time. Performance: compared to normal expectations (work functioning)
Health and Performance Questionnaire (HPQ)	Deckersbach et al. (2010), Duarte et al. (2016)	WP and WF	HPQ is a self-report questionnaire in which the patient can rate their own performance on a scale. Next to work performance, absenteeism and job-related accidents are recorded
Quality of Life Enjoyment and Satisfaction Questionnaire (Q-LES-Q)	Latalova et al. (2010)	WP and WF	Participation: whether the client works or not. Functioning: satisfaction and experience with several aspects of working
Multnomah Community Ability Scale (MCAS)	Lewandowski et al. (2020)	WF	MCAS was developed to assess functioning in patient populations across multiple domains including independence in daily living, social interest and involvement, work and leisure activities, and participation in treatment

Various instruments for measuring work participation were included in the studies. In total, 12 instruments to measure work participation were included. These instruments ranged from questionnaires that examined participation to some extent to categorizations of occupational attainment. Most employed in studies was the Work and Social Adjustment Scale (WSAS; Mundt et al. 2002) in four studies.

For work functioning, 13 different measurements were included in the studies. Most used were the Global Assessment of Functioning, GAF (Endicott et al. 1976), in 20 of the studies, and the Functioning Assessment Short Test, FAST (Rosa et al. 2007), in 15 of the studies. Other measurements to examine work functioning included the Social and Occupational Functioning Assessment Scale, SOFAS (Goldman et al. 1992), and the Social Adjustment Scale, SAS (Weissman and Bothwell 1976). Studies that used performance-based assessments, such as the UCSD Performance-Based Skills Assessment, UPSA (Patterson et al. 2001), provide a more detailed examination of the quality of functioning. One study (Bowie et al. 2010), utilized this performance-based measurement and an observational measure in addition to questionnaires.

Four of the included instruments measured both work participation and functioning, such as Multidimensional Scale of Independent Functioning, MSIF (Jaeger et al. 2003), which reports on role functioning and performance (Jaeger et al. 2003) and the Life Functioning Questionnaire, LFQ (Altshuler et al. 2002), which assesses the degree of experienced difficulties and participation at work. Besides measurements that were already developed, in some studies a questionnaire was specifically developed for the purpose of the study (Martinez-Aran et al. 2007; Bonnin et al. 2014; Levy et al. 2013; Strassnig et al. 2017; Robertson 2006; Crowe et al. 2020; DeTore et al. 2018; Lee et al. 2015; Boland et al. 2015). Most of these studies aimed to provide a description of work participation. For example, DeTore et al. (2018) measured how many months a patient was employed in the competitive job market, while Boland et al. (2015) measured the total months of employment, number of firings, and number of self-terminated employment.

#### Measuring executive functions

In most (74 of 82) of the studies EF were measured using broader test batteries including also other aspects of cognitive functioning. In 11 studies, cognitive functioning was evaluated using a subjective measure, either self-report or interview based (Altshuler et al. 2007; Deckersbach et al. 2010; Luo et al. 2020; Jiménez et al. 2012; Martinez-Camarillo et al. 2019; Robertson 2006; Crowe et al. 2020; Gilbert et al. 2010; Jensen et al. 2015; Samalin et al. 2016; O'Donnell 2016). In one of the studies event-related potentials (P300) were measured along with more traditional tests (Kaya et al. 2007). One of the included studies measured self-regulation, namely with a semi-structured interview (Robertson 2006). Of the included studies, 40 gave no rationale for the used measurements. Another 34 did give a reason for including certain measures, mostly because measures were shown to be valid and reliable with the BD population, to ensure replication by using widely employed batteries, and cognitive tasks that are shown to be associated with frequently occurring deficits in BD. Four studies (Lewandowski et al. 2020; Rosa et al. 2014; Sanchez-Autet et al. 2018; Liu et al. 2021) mention the recommendations made by the International Society for Bipolar Disorders (ISBD) regarding cognitive assessments (Miskowiak et al. 2017; Yatham et al. 2010). The included measurements are summarized in Table 3.

**Table 3:** Measurements for executive functions

Measurement	Used in	Description measurement
BIS-11	Jiménez et al. (2012), O’Donnell (2016)	BIS-11 is a self-rated 30-item questionnaire which has three subscales: attentional/cognitive, which measures tolerance to cognitive complexity and persistence; Motor, which measures the tendency to act on the spur of the moment; and Non-planning impulsivity, which measures the lack of sense of the future
The Controlled Oral Word Association Test (COWAT-FAS); category fluency	Anaya et al. (2016), Baune et al. (2013), Bearden et al. (2011), Bello (2009), Boland and Alloy (2013), Bowie et al. (2010), Burdick et al. (2010), Chen et al. (2021), Crouse et al. (2020), Demant et al. (2015a, b), Depp et al. (2012b), Drakopoulos et al. (2020), Godard et al. (2011), Esan et al. (2020)^a^; Jensen et al. (2015, 2016), Latalova et al. (2010), Lee et al. (2013, 2015), Levy et al. (2013), Lewandowski et al. (2020); Lomastro et al. (2020), Luo et al. (2020), Martinez-Aran et al. (2007), Miguélez-Pan et al. (2014), Mora et al. (2013), Mur et al. (2008, 2009), O’Donnell (2016), O’Donnell et al. (2017), Olley et al. (2005), Ryan et al. (2013), Sachs et al. (2020), Simonsen et al. (2010); Solé et al. (2012, 2018), Strassnig et al. (2017), Tabarés-Seisdedos et al. (2008), Torrent et al. (2007, 2013), Varo et al. (2017), Wingo et al. (2010), Zubieta et al. (2001), Zyto et al. (2016)	The Controlled Oral Word Association Test, is considered to be a measure of spontaneous word fluency and is believed to be subserved by executive or prefrontal cortical functioning
Delis–Kaplan executive function system (D-KEFS)	Drakopoulos et al. (2020)	Assesses key components of executive functions within verbal and spatial modalities
The Inter-Dimensional/Extra-Dimensional Shift (ID/ED Shift), Stockings of Cambridge (SOC), (CANTAB)	Lee et al. (2015), Olley et al. (2005)	The Inter-Dimensional/Extra-Dimensional Shift (ID/ED Shift), a test of abstract problem solving and attentional set-shifting, is similar to the widely used Wisconsin Card Sort Test (WCST) The Stockings of Cambridge (SOC) task, a complex problem solving and planning task akin to the Tower of London
Executive Interview (EXIT)	Altshuler et al. (2007)	The EXIT is a 15-min structured interview that correlates well with other measures of executive cognitive function and can be administered by lay personnel. The EXIT contains items that tap into a variety of executive cognitive domains, including perseveration, response set-switching, generation de novo of stories, generation of word lists, and executing tasks during interference
Stroop Colour–Word Interference Test (SCWT)	Anaya et al. (2016), Baune et al. (2013), Bonnín et al. (2010, 2014), Dickerson et al. (2010), Kaya et al. (2007), Liu et al. (2021), Luo et al. (2020), Martinez-Aran et al. (2007), Miguélez-Pan et al. (2014), Mora et al. (2013), Mur et al. (2008, (2009), O’Donnell (2016), O’Donnell et al. (2017), Olley et al. (2005), Rosa et al. (2014), Ryan et al. (2013), Simonsen et al. (2010); Solé et al. (2012, 2018), Strassnig et al. (2017), Tabarés-Seisdedos et al. (2008), Torrent et al. (2007, 2013), van Rheenen and Rossell (2014), Varo et al. (2017), Vierck and Joyce (2015), Zubieta et al. (2001), Zyto et al. (2016)	The Stroop Colour–Word Interference Test is a test of cognitive inhibition
TMT-B	Baune et al. (2013), Bearden et al. (2011), Bello (2009), Bonnín et al. (2010), Bonnín et al. (2014), Bowie et al. (2010), Burdick et al. (2010), Crouse et al. (2020), Demant et al. (2015a, b), Dickerson et al. (2004, 2010), Drakopoulos et al. (2020), Forcada et al. (2015), Fulford (2011), Latalova et al. (2010), Lee et al. (2013), Levy et al. (2013), Lomastro et al. (2020), Luo et al. (2020), Martinez-Aran et al. (2007), Miguélez-Pan et al. (2014), Mora et al. (2013), Mur et al. (2008), Mur et al. (2009), O’Donnell (2016), O’Donnell et al. (2017), Ryan et al. (2013), Solé et al. (2012, 2018), Strassnig et al. (2017), Tabarés-Seisdedos et al. (2008), Torrent et al. (2007, 2013), van Rheenen and Rossell (2014), Varo et al. (2017), Wilder-Willis (2001), Wingo et al. (2010), Zyto et al. (2016)	The Trail Making Test B or Trails B is considered a task of visual search, visuospatial sequencing, and cognitive set shifting
Digit Span (DS) subtest of subtest of Wechsler Adult Intelligence Scale	Anaya et al. (2016), Boland and Alloy (2013), Boland et al. (2015), Bonnín et al. (2010), Chen et al. (2021)^b^; Depp et al. (2012b), Leany (2011), Luo et al. (2020), Mora et al. (2013), Mur et al. (2008), O’Donnell (2016), O’Shea et al. (2010), Zyto et al. (2016)	Digit Span involves attentional processes of being able to hold sequences of strings of numbers in working memory and reiterate the sequences in the auditory channel
Letter Number Sequencing (LNS) subtest of Wechsler Adult Intelligence Scale	Anaya et al. (2016), Bearden et al. (2011), Bowie et al. (2010), Demant et al. (2015b), Depp et al. (2012b), Dickerson et al. (2010), Jensen et al. (2015, 2016), Lewandowski et al. 2020; Rosa et al. (2014), Strassnig et al. (2017), Torrent et al. (2013), Varo et al. (2017), Wingo et al. (2010)	The pt is read a series of letters and numbers and is required to repeat them back with the letters in alphabetical order and the numbers in numerical order. The test was designed to measure an individual’s ability to hold verbal information in memory while he/she manipulates it
Parametric GO/NO-GO test	O’Donnell (2016), O’Donnell et al. (2017), Ryan et al. (2013)	The PGNG consists of three levels of difficulty assessing attention, set shifting, and processing speed, with the two more difficult levels assessing inhibitory control
WMS-III Spatial Span Subtest	Leany (2011), Lewandowski et al. (2020)	The SST measures an individual’s ability to hold a visual spatial sequence of locations in working memory and reproduce the sequence, thereby being a measure of visual working memory
TOL	Boland et al. (2015), Chen et al. (2021), Drakopoulos et al. (2020), Godard et al. (2011), Miguélez-Pan et al. (2014)	The TOL assesses spatial planning by given rules
WCST	Bello (2009), Bonnín et al. (2014), Bonnín et al. (2010), Bowie et al. (2010), Burdick et al. (2010), Depp et al. (2012b), Dickerson et al. (2010), Leany (2011), Levy et al. (2013), Lomastro et al. (2020), Martinez-Aran et al. (2007), Miguélez-Pan et al. (2014), Mora et al. (2013), Mur et al. (2008), Mur et al. (2009), O’Donnell (2016), O’Donnell et al. (2017), Ryan et al. (2013), Sachs et al. (2020); Solé et al. (2012), Solé et al. (2018), Tabarés-Seisdedos et al. (2008), Torrent et al. (2007, 2013), Varo et al. (2017), Wilder-Willis (2001), Zubieta et al. (2001), Zyto et al. (2016)	A test of executive functioning ability that requires subjects to sort cards, altering the chosen sorting approach based on feedback received at unannounced intervals during the task

^a^Subtest SKIP

^b^Subtest BAC-A

The most common way to assess executive functions in the included studies was a combination of traditional and widely used tests such as TMT-B (Reitan 1958), for switching/divided attention, Wisconsin Card Sorting Test (Heaton 1981) for mental flexibility, Stroop-Color Word Test (Golden 1978), for inhibition/selective attention, COWAT letter & categorical fluency (Patterson 2018), Digits Span and Letters and Numbers Sequencing from the WAIS (Wechsler 2008), Spatial Working Memory test from the CANTAB (Robbins et al. 1994) or the Purdue Pegboard test (Tiffin and Asher 1948), for measuring working memory (Wingo et al. 2009, 2010; Burdick et al. 2010a; Martinez-Aran et al. 2007; Lomastro et al. Nov 2020; Bonnin et al. 2014; Bowie et al. 2010; Drakopoulos et al. 2020; Duarte et al. 2016; Levy et al. 2013; Luo et al. 2020; Solé et al. 2018; Strassnig et al. 2017; Tabarés-Seisdedos et al. 2008; Torrent et al. 2007, 2013; Boland and Alloy 2013; Depp et al. 2012b; Varo et al. 2017; Demant et al. 2015a; Rosa et al. 2014; Liu et al. 2021; Sachs et al. 2020; Anaya et al. 2016; Bearden et al. 2011; Bonnín et al. 2010; Demant et al. 2015b; Dickerson et al. 2004, 2010; Forcada et al. 2015; Lee et al. 2013; Mora et al. 2013; Jensen et al. 2016; Simonsen et al. 2010; Zubieta et al. 2001). Five studies (O'Donnell 2016; Ryan et al. 2013; O'Donnell et al. 2017; O'Shea et al. 2010; Zyto et al. 2016) combined these tests with either a parametric Go/No-Go test (Langenecker et al. 2007), BADS (Wilson et al. 1996), Haylings Sentence Completion Task (Burgess and Shallice 1997) or the Tower of London (Delis et al. 2001). One study (Drakopoulos et al. 2020) used the whole of D-KEFS (Delis et al. 2001) a test battery comprising executive functioning, consisting of many of the traditional executive measures described above. Five studies (Deckersbach et al. 2010; Demant et al. 2015a; Dickerson et al. 2004; Esan et al. 2020; Chen et al. 2021) used screening tools and test batteries in which tests for EF are embedded to a limited extent such as the Screen for Cognitive Impairment in Psychiatry, SCIP (Purdon 2005), the BAC-A (Barker et al. 1994) and the Repeatable Battery of the Assessment of Neuropsychological Status, RBANS (Randolph 1998).

Two studies (Altshuler et al. 2007; Deckersbach et al. 2010) used self-rating scales for subjective executive problems such as the Frontal Systems Behavior Rating Scale, FrSBe (Grace and Malloy 2001), and the Executive Interview, EXIT (Royall et al. 1992). Five studies (Luo et al. 2020; Martinez-Camarillo et al. 2019; Demant et al. 2015a; Jensen et al. 2015; Zyto et al. 2016) used a self-rating scale to inquire about general cognitive complaints such as the Massachussetts General Hospital Cognitive and Physical Functioning Questionnaire, CPFQ (Fava et al. 2009), the Cognitive Failure Questionnaire, CFQ (Broadbent et al. 1982), or the Cognitive Complaints in Bipolar Disorder Rating Scale, COBRA (Rosa et al. 2013). Other self-ratings used for general cognition consisted of self-designed formats in relation to daily functioning (Gilbert et al. 2010) or a visual analogue scale for the patients’ perception of cognitive functioning (Samalin et al. 2016).

Two studies (Robertson 2006; Crowe et al. 2020) examined the subjective experiences with cognitive problems in daily life of patients with BD and used a semi-structured interview to do this. Robertson (2006) was the only study that inquired about self-regulation. Work-motivation was found to be measured with the Motivation for Work Questionnaire, MWQ (Colis and Galilea 1995), by Martinez-Camarillo et al. (2019). Two studies (Jiménez et al. 2012; O'Donnell 2016), used the Baratt Impulsivity Scale, BIS-11 (Barratt et al. 1983), to measure self-reported impulsivity.

### Neurocognitive functions, clinical variables and occupational functioning

Besides EF, other neurocognitive functions have been found to be associated with occupational functioning in BD. A comprehensive review by Duarte et al. (2016) including 23 papers showed the EF, followed by verbal memory, processing speed and attention, to be the most common neurocognitive functions to be associated in the relationship between executive functioning and occupational functioning in BD. This is corroborated by the studies included in this review, of which five did not find a relationship that included EF but rather memory and attention (Sanchez-Moreno et al. 2009; Wilder-Willis 2001; Andreou and Bozikas 2013; Latalova et al. 2010; Vierck and Joyce 2015). In a cross-sectional study with 47 participants (Bello 2009), better work functioning was only related to visual memory and not to other domains including EF. Furthermore, Duarte et al. (2016) pointed out several clinical and illness variables to be associated with occupational function such as premorbid IQ, residual depression, BD diagnoses, medication, a history of psychosis, number of hospitalizations and substance abuse. A cross-sectional study by Jiménez et al. (2012) including 138 persons, found a relation between manic and depressive symptoms, prior hospitalizations, total number of mood episodes, impulsivity and current occupational functioning.

In a longitudinal study by Dickerson et al. (2010) occupational status was not significantly associated with any cognitive variables at 6-month follow-up after a psychiatric hospital admission for a mood episode. Only limited recovery of occupational role had taken place at 6-month follow-up, 54% were working full-time in a competitive job or studying, whereas almost all were working or studying full-time before admission. Bearden et al. (2011) found working memory and processing speed to predict occupational recovery after admission for an episode of mania. In a longitudinal study by Crouse et al. (2020) data-driven neurocognitive subgroups consisting of 629 young adults with emerging mental disorder (BD = 14%), with different degrees of impairment, were followed for 3 years to investigate whether the distinct groups were associated with different social and occupational trajectories. This study showed that the globally impaired subgroup (z-scores − 1 to − 2 sd, flexibility and set shifting being the most impaired), of which 11% BD, showed the poorest course of social and occupational functioning regardless of gender, premorbid IQ and educational level, and symptom severity.

More than half of the papers assessed patients in an (relatively) euthymic state (Martinez-Aran et al. 2007; Mur et al. 2008, 2009; Bello 2009; Bonnin et al. 2014; Deckersbach et al. 2010; Luo et al. 2020; Solé et al. 2012, 2018; Torrent et al. 2007, 2013; Wilder-Willis 2001; Fulford 2011; Jiménez et al. 2012; Martinez-Camarillo et al. 2019; Miguélez-Pan et al. 2014; Varo et al. 2017; Boland et al. 2015; Jensen et al. 2015; Samalin et al. 2016; Kaya et al. 2007; Rosa et al. 2014; Liu et al. 2021; Sachs et al. 2020; Anaya et al. 2016; Bearden et al. 2011; Bonnín et al. 2010; Dickerson et al. 2010; Forcada et al. 2015; Mora et al. 2013; Zubieta et al. 2001; Ryan et al. 2013; O'Shea et al. 2010; Zyto et al. 2016; Esan et al. 2020; Chen et al. 2021; Latalova et al. 2010; Leany 2011; Olley et al. 2005; Rheenen and Rossell 2014). In around 1/5 the mood state was not reported, and the influence of mood was not established (Levy et al. 2013; Sanchez-Moreno et al. 2009; Strassnig et al. 2017; Tabarés-Seisdedos et al. 2008; Robertson 2006; Crowe et al. 2020; DeTore et al. 2018; Gilbert et al. 2010; O'Donnell 2016; Simonsen et al. 2010; O'Donnell et al. 2017). Another fifth of the reviewed papers investigated groups with mixed moods (Wingo et al. 2009, 2010; Altshuler et al. 2007; Bowie et al. 2010; Lewandowski et al. Apr 2020; Demant et al. 2015a; Sanchez-Autet et al. 2018; Demant et al. 2015b; Jensen et al. 2016; Crouse et al. 2020; Rheenen and Rossell 2014; Burdick et al. 2010). A small number included clinically depressed groups (Burdick et al. 2010a; Godard et al. 2011).

We have divided the original research included in this review into two categories based on study design and subsequent results: (1) studies in which associations between EF and occupational functioning in BD patients are explored, and (2) studies that examine the predictive value of EF on occupational functioning. Below we present the findings regarding these two types of results. We differentiate between results regarding work participation and results on work functioning.

#### Associations between EF and occupational functioning

Of the 39 cross-sectional studies that used objective measures for executive functions, 24 showed a specific association between EF and occupational functioning. However, not all studies reported the results of EF tests separately but rather in a composite score with other cognitive functions. Besides objective test batteries, subjective measurements were employed to examine neurocognitive functioning. Of the 44 cross-sectional studies, four included a subjective measure of general cognitive function and two a specific subjective measure of EF in relation to occupational functioning. Additionally, one qualitative study was included in this review, in which the experiences of patients regarding cognitive impairment were studied (Crowe et al. 2020). Table 4 summarizes 26 papers in which an association between aspects of EF and occupational functioning was found together with an appreciation of the strength and direction of the association. Most of the studies (22) found a small to medium effect.

**Table 4:** Associations between EF and occupational functioning

Aspects of EF					
Aspects of OF ⇒	Employment	Workperformance/adjustment	Skilled job	Attendance	Lifetime firings
Working memory (DS, LNS, spatial span)		Jensen(2015)S+ Lee(2013)M+ Sanchez-Autet^a^(2018)S+			
Planning (TOL)			Miguelez-Pan(2014)S+		Boland(2015)M+
Set shifting (TMT-B)	Depp(2012)M+ Sole(2012)M+ Sole(2018)M+	Lee(2013)M +	Miguelez-Pan(2014)S+		
Inhibitory control/interference (SCWT, PGNG)	Mur (2009)L+	Zubieta(2001)M+			Boland(2015)L+
Impulsivity (Bis-11)		Jimenez(2012)^b^S−			
Cognitive flexibility (WCST, ID/ED shift)	Sole(2018)M+	Bonnin(2014)S+ O’Donnell (2016/2017)S+ Olley (2005)M+		O’Donnell (2016/2017)S+	
Fluency (COWAT-FAS, categories)	Godard(2011)M+ Martinez-Aran(2007) S+				
Emotion regulation/motivation	Van Rheenen(2014)S+ Martínez-Camarillo^c^ (2019)	Samalin(2016)S+			
Self-regulation		Robertson (2006)^c^			
Subjective EF (EXIT)	Altshuler(2007)M+				
Composite EF (WCST, fluency, TMT-B, PGNG^d^, emotion-processing^d^)	Drakopoulus (2020)M/L+ Ryan(2013)^d^M+	Lomastro (2020)L+			

*S* small ES, *M* medium ES, *L* large ES

+/−: Direction of association

^a^Only women

^b^+ Impulsivity→ − WF

^c^No ES could be calculated

A poorer set shifting ability (Stroop switching) and planning deficits (TOL) have been shown to be associated with more life time firings in BD (Boland et al. 2015) whereas a good planning ability (TOL) and a good ability to switch/divided attention (TMT-B) was associated with holding a skilled job (Miguélez-Pan et al. 2014). Bonnin et al. (2014) found perseverative errors/poor set shifting (WCST) together with manic episodes to contribute to poor work adjustment, explaining up to 36% of the variance in work adjustment, in which manic episodes was the strongest factor. Altshuler et al. (2007) found the EXIT score, number of psychotropic medications and number of psychiatric hospitalizations to be the most significant factors to explain occupational role functioning. In a cross-sectional study with 120 patients (Drakopoulos et al. 2020) found that general executive functions (D-KEFS) were stronger determinants of occupational functioning than general IQ and illness severity. Jensen et al. (2016) identified discrete subgroups based on the neurocognitive functioning in a large group of fully or partially remitted mixed BD I and BD II of which the globally impaired group had the worst work outcome.

In a study by Demant et al. (2015a) only subjective cognitive complaints were associated with poor social and occupational functioning, whereas objective cognitive functioning did not. In a study by Gilbert et al. (2010), similarly, subjective cognitive functioning showed a significant correlation with employment status, but physician-rated cognitive problems did not. Deckersbach et al. (2010) did not find any correlation between neuropsychological measures and work impairment in a small sample with moderate to severe degree of work impairment. However, changes in EF, in part, did account for improvements in occupational functioning after a functional remediation treatment. In a study by Dickerson et al. (2004) only verbal memory was related to work status, however this study used only a limited number of EF test. In a study by Bowie et al. (2010) neurocognition’s association with working skills were entirely mediated by adaptive and social skills, comprising among others, everyday living skills, motivation, social competence, and meta-cognition.

#### Predictive value of EF on occupational functioning

As to the predictive value of EF, 19 longitudinal studies were included in which predictors of occupational functioning were examined (Burdick et al. 2010a; Levy et al. 2013; Mur et al. 2008; Strassnig et al. 2017; Tabarés-Seisdedos et al. 2008; Robertson 2006; Lee et al. 2013, 2015; O'Donnell 2016; Bearden et al. 2011; Bonnín et al. 2010; Dickerson et al. 2010; Mora et al. 2013; O'Donnell et al. 2017; Chen et al. 2021; Crouse et al. 2020; Leany 2011). Nine of these longitudinal studies found that EF, among other neurocognitive functions, are predictive of occupational functioning at follow-up. Table 5 summarizes these papers and gives an appreciation of the strength and direction of the longitudinal associations between aspects of EF and occupational functioning. More than half of the studies showed this association to be large, mostly related to working memory, set-shifting and interference.

**Table 5:** Predictive value of EF on occupational functioning (longitudinal)

Aspects of EF				
Aspects of OF ⇒	Employment	Workfunctioning/adjustment	Attendance	Occupational recovery
Working memory (DS, LNS, spatial span)		Bonnin(2010)L+ Lee(2013)L+		Bearden(2011)L+
Set shifting (TMT-B)	Mora(2013)^a^	Lee(2013)L+		
Inhibition/interference (SCWT)	Mur (2008)L+ Mora(2013)^a^			
Reasoning/cognitive flexibility (WCST, ID/ED shift)		Bearden(2011)M+ O’Donnell(2016)S+	O’Donnell(2016)S+	
Emotion-regulation/motivation		Robertson (2016)^a^		
EF composite score		Tabarés-Seisdedos(2008)M+		

*S* small ES, *M* medium ES, *L* large ES

+/−: Direction of association

^a^No ES

Most longitudinal studies focused on work participation, in which the employment status (e.g. working, yes or no) of participants was related to outcomes on measures of neurocognition, including EF. Almost all studies focusing on this aspect of occupational functioning found EF to be predictive of this domain. For example, a longitudinal study with 32 subjects conducted by Bonnin et al. (2010) showed working memory, in addition to subdepressive symptomatology, to be the strongest associated with occupational functioning 4 years later. This finding is consistent with a later cross-sectional and longitudinal study by Bearden et al. (2011), in which baseline cognitive changes in all domains, except for processing speed, were highly significant predictors of occupational recovery. At the time of symptomatic recovery, the domains of working memory/attention and processing speed were strongly associated with concurrent occupational recovery. These results are further corroborated by the included reviews and meta-analyses. In a systematic review Baune et al. (2013) found verbal learning and memory, processing speed, attention, and EF to be prospectively associated with occupational functioning in several longitudinal studies. In a second review by Baune and Malhi (2015) EF and reaction time were associated with active occupation after 2 years, whereas lower EF and processing speed was predictive of a lower occupational functioning at follow up after 6 years. One study showed that neither memory or EF were predictive of functional outcomes (Dickerson et al. 2010).

Regarding work functioning, two longitudinal studies report that EF are associated with this aspect of occupational functioning (O'Donnell et al. 2017; Leany 2011). For example, O'Donnell et al. (2017) report that deficits in cognitive flexibility were predictive of more difficulties in overall work functioning, quality of work, and lower work performance.

#### Self-regulation, emotion regulation and occupational functioning

Seven studies examined self-regulation or an aspect thereof, including emotion regulation, in relation to occupational functioning (Fulford 2011; Martinez-Camarillo et al. 2019; Varo et al. 2017; Robertson 2006; DeTore et al. 2018; Ryan et al. 2013; Rheenen and Rossell 2014). Five of these studies focused on aspects of emotion, emotion regulation, and emotional intelligence. Ryan et al. (2013) found that work status was predicted by emotion processing, and Varo et al. (2017) found that patients characterized with low performance on the Mayer-Salovey-Caruso Emotional Intelligence Test (MSCEIT) experienced the most impairments in overall functioning and autonomy as measured with the FAST. Additionally, Fulford (2011) found that the Managing Emotions scale of the MSCEIT was related to occupational prestige and job stability. Van Rheenen and Rossell (2014) found that emotion regulation was the strongest predictor of objective functioning in patients with bipolar disorder. However, emotion regulation appeared to primarily influence mood symptomatology, which in turn impacts the functioning of patients.

Regarding self-regulation, the study conducted by Robertson (2006) is a case-study that focused on the development of self-regulation over time, in which occupational therapy supported the client to develop self-regulation, which led to better functioning during work. The study by Martinez-Camarillo et al. (2019) examined work motivation, which was impacted by subjective cognitive complaints and unemployment in this study.

### Intervention studies

Our search strategy identified four studies in which a cognitive or functional remediation program was evaluated and occupational functioning was one of the examined parameters (Deckersbach et al. 2010; Demant et al. 2015b; Torrent et al. 2013; Zyto et al. 2016). Despite the limited number of studies, the results in this area show promising effects in reducing the impact of cognitive dysfunction (including EF deficits) on daily functioning.

In three of the four included papers, an improvement in occupational functioning was observed at follow-up (Deckersbach et al. 2010; Torrent et al. 2013; Zyto et al. 2016). Deckersbach et al. (2010) report that changes in EF partially account for these improvements, and that presenteeism improved more than absenteeism. Torrent et al. (2013) report that several patients who received functional remediation were able to attain employment or improve their occupational functioning, Zyto et al. (2016) also report that patients were able to get a job after receiving functional remediation or improve their occupational functioning. Demant et al. (2015b) reported no effects of the remediation program on the several outcome measures of the study, including EF and occupational functioning. However, this may be due to the short-term nature of the intervention, and the inclusion of participants who did not demonstrate objective impairments in cognition.

## Discussion

**Fig. 3.**
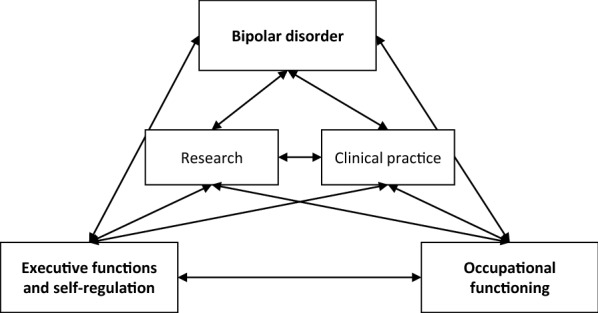
Relationships between BD, OF and EF

The aim of this review was to gain more insight in the relationships between executive functioning (EF)—viewed as core mechanisms of self-regulation—and occupational functioning in patients with BD. The last decade has seen an increase in research addressing these three themes and how they relate to each other, which has advanced our understanding of these relationships considerably. Figure 3 schematically shows the various relationships possible between the three main themes (in bold) of this review and how research and clinical practice may influence these. In this scoping review we reviewed 82 papers, which all discussed the relationships to some extent. Below we discuss our findings regarding the three themes (bipolar disorder, occupational functioning, and executive functions and self-regulation) and provide recommendations for both research and clinical practice.

### Main findings

The main finding of this scoping review is that EF are an important neurocognitive domain associated with impairments in occupational functioning in patients with BD, both in cross-sectional and longitudinal studies. Relationships between EF and occupational functioning were established in several studies, and a recent study showed EF to be more predictive of occupational functioning than general IQ (Drakopoulos et al. 2020). Of the 26 cross-sectional studies that showed an association between EF and occupational functioning 22 showed small/medium effects. The included reviews and meta-analyses further elaborate on the main finding of the current scoping review. Results from several regularly used EF tests were found to be associated with occupational functioning and to be predictive of occupational functioning for up to 6 years. EF that seem the most predictive of occupational functioning are working memory, set shifting and inhibition/interference. For these functions the associations appear the strongest, with relatively large effect sizes. Considering the influence of mood symptoms, most patients in the reviewed studies were euthymic, however mostly meaning that many still had subsyndromal depressive symptoms. Some studies found a relationship with subsyndromal depressive symptoms and cognitive and functional impairments (Bonnin et al. 2014; Anaya et al. 2016). But even when controlling for the depressive symptoms, cognitive impairments, especially in working memory and processing speed, are associated with occupational role recovery (Bearden et al. 2011). There are many differences between the applied definitions, measurements, and subsequent results. Furthermore, EF are not the only neurocognitive functions essential to occupational functioning. Other neurocognitive functions implicated in occupational impairments are processing speed, verbal memory, and attention. In addition, the precise nature of the relationship between EF and occupational functioning remains unclear. Implicated in most studies is the hypothesis that impairments in EF are responsible for impairments in occupational functioning. However, there has not been extensive research regarding the effects of employment on EF, and whether employment may have a protective or even stimulating effect on EF. Research regarding cognitive reserve, such as the study by Anaya et al. (2016), may provide more insight into this relationship since employment is viewed as one of the factors that increases cognitive reserve.

We have reviewed four studies on cognitive or functional remediation programs in which occupational functioning was included as parameter. The results show that occupational functioning may be improved after following a cognitive or functional remediation program, sometimes as a (partial) result of improved EF. Therefore, these programs are promising interventions for occupational recovery.

### Neurocognition, executive functions, and self-regulation

There is a long tradition regarding research and the conceptualization of EF, yet there is still no definitive consensus on the precise nature of these neurocognitive functions (Barkley 2012; Cramm et al. 2013; Baggetta and Alexander 2016). There is, however, a golden standard in neuropsychological research, which consists of test batteries and instruments commonly used to measure objective neurocognitive impairment. Furthermore, for research regarding cognitive impairment in BD, two papers have been published with recommendations by the International Society for Bipolar Disorders (ISBD) regarding cognitive assessments (Miskowiak et al. 2017; Yatham et al. 2010). Of the studies included in this scoping review, only four studies mention these recommendations (Lewandowski et al. 2020; Rosa et al. 2014; Sanchez-Autet et al. 2018; Liu et al. 2021). Furthermore, considering the ongoing scientific discussion regarding the nature of EF, it is noteworthy that none of the included papers mention an underlying theoretical framework or rationale for implementing certain measurements besides the current golden standard in neuropsychological research.

Regarding self-regulation, theoretical and conceptual literature is increasingly describing EF as essential for the self-regulation ability (Snyder et al. 2015; Nigg 2017; Hofmann et al. 2012). Contrary to our expectations, we found no mention of this conceptual link in the included studies. Only one study examined self-regulation, all other studies used common neuropsychological tests, standardized interviews or self-report questionnaires about general cognitive functioning or EF specifically. Furthermore, five of the included studies examined emotion regulation or related concepts such as emotional intelligence, which can be viewed as an aspect of self-regulation and is of particular interest for BD. These studies show that emotion related processes, such as emotional intelligence and emotion regulation, are associated with and predictive of occupational functioning. However, emotion regulation may impact occupational functioning by influencing mood symptomatology (Rheenen and Rossell 2014).

Some variations in cognition are due to a younger population, early in the course of the illness trajectory with sometimes marginal deficits in cognition and slight problems in occupational functioning. This points out the importance of defining and taking subgroups of levels of neurocognitive functioning into account. This was shown in the study by Crouse et al. (2020) where patients were divided into subgroups according to results from a cluster analysis of neurocognitive functioning, resulting in three subgroups. Each subgroup is strongly associated with a different occupational trajectory beyond IQ, educational level, and symptoms. In addition, the study by Rosa et al. (2014) assessed functional impairment and cognitive functioning according to a staging model as proposed by Kapczinski et al. (2009). The results from this study indicate that categorizing patients on a continuum of disorder progression may be meaningful for providing treatment that is attuned to the individual needs of patients.

### Occupational functioning

One of the main themes of focus of this scoping review was occupational functioning; a construct that contains the extent to which individuals participate in the job market, as well as the quality of work functioning and needed skills. While some papers included a definition or description of occupational functioning, there was an overall lack of conceptual clarity. The definitions of occupational functioning ranged from categorical definitions of being employed or unemployed, absenteeism versus presenteeism, to more descriptive definitions such work skills, adaptive skills or level of supervision needed. Surprisingly, none of the studies investigated directly if a participant worked in conformity to his or her level of education. Measurements used for occupational functioning differed greatly, an observation already stated in a recent systematic review (Chen et al. 2019).

Most included studies have not reported on contextual factors which may influence the occupational functioning of patients with BD. Whether patients have good opportunities to participate in the competitive job market depends not only on their own capabilities, but also on societal factors (macro level) such as employment opportunities, access to effective vocational rehabilitation programs, stigma towards mental illness, possibility to receive a disability pension or participate in sheltered work. Most studies were published in North America, Australia, and Europe, possibly pointing towards a western bias within this specific field of research. Regarding the meso level, most studies included in this review focused on the patient population and, when applicable, neurocognitive subgroups. Considering the heterogeneous nature of bipolar disorder, specifying subgroups based on neurocognitive performance may lead to more specific results, interventions, and prognoses. Lastly, the micro level is less pronounced in the included research. A focus on individual neurocognitive testing is mostly conducted in individual treatment trajectories and interventions. However, important contextual factors on micro level receive little attention within research. For example, the work environment, nature of relationships with colleagues, and specific skills needed for the work are contextual factors on the micro level, which may impact the occupational functioning of patients greatly. Considering the work functioning aspect of occupational functioning, a focus on the quality of functioning of individual patients or patient groups (meso level) could further clarify the relationship between specific EF or other neurocognitive functioning and real-world work functioning. Measurements used by occupational therapists, such as the Perceive, Receive, Plan and Perform (PRPP; Chapparo and Ranka 2012), may be useful for relating cognitive deficits to how patients function within their daily context. Furthermore, most neuropsychological tests are administered in structured situations, possibly underestimating the role of EF in real-world functioning where less structure calls for shifting and handling of much more stimuli at once.

### Recommendations and implications for research and clinical practice

The findings from this scoping review show that, while there are many studies that examine the relationships between EF, occupational functioning, and bipolar disorder, there are various gaps in the current knowledge. Research and clinical practice are of importance for further advancing our knowledge of these relationships and how individuals with BD can be supported in their recovery process.

As described in the results and discussion, definitions regarding EF and occupational functioning are mostly lacking in the included studies. Where definitions were given, there was no apparent consensus. A clear theoretical framework to relate neurocognitive deficits and self-regulation to occupational functioning in BD can support future research considerably in defining the constructs and choosing relevant measurements. The precise nature of the relationships between EF, occupational functioning, and BD is currently still unclear. A theoretical framework can support further examination of these relationships and the underlying mechanisms. For example, identifying how and why (subdomains of) EF can be so influential on occupational functioning may help advance treatment practices and transfer of skills learned in remediation programs. Furthermore, considering the importance of affective states, it may be important to incorporate emotions and emotion regulation in such a theoretical framework, as opposed to only describing ‘cool’ neurocognitive functions.

The results of our review show that discriminating between subgroups based on neurocognitive functioning may further clarify associations between neurocognitive deficits and employment trajectories in patients with BD. Studies that focus on neurocognitive subgroups are scarce and we recommend a more precise focus on neurocognitive performance when examining the relationship between neurocognition and occupational functioning in bipolar disorder. Staging models, such as the one proposed by Kapczinski et al. (2009), may be useful to allocate patients to subgroups based on aspects of illness progression and experienced impairments. The usefulness of allotting patients to certain subgroups also extends to intervention development and research. Demant et al. (2015b) report that their functional remediation program might have been insufficient for the included patients due to the short-term sessions and inclusion of patients without objective cognitive impairments. Attuning functional remediation to the individual needs of patients, for example by increasing the number of sessions or adding individual sessions, may be more beneficial for certain subgroups of patients. Acknowledging neurocognitive subgroups and their predictive value regarding occupational trajectories, interventions could become more efficient by targeting the right level of functioning.

Despite the gaps in our current knowledge and lack of proven effective interventions, clinical practice may already benefit from the findings of this review. Training cognitive skills, especially in the context in which the individual patient needs these skills, may benefit occupational functioning. Functional remediation programs show promising results in improving occupational functioning in this regard. Furthermore, research in other psychiatric populations show that cognitive remediation combined with vocational rehabilitation methods such as Individual Placement and Support are a promising combination for promoting the transfer of skills (Duin et al. 2021). Patients with BD may benefit from this integrative approach to neurocognitive and occupational impairment. We recommend examining and reporting contextual factors on micro, meso, and macro level, since these factors can be of great impact on the occupational and neurocognitive functioning of patients with bipolar disorder. Furthermore, when designing interventions, more descriptive and qualitative measures of occupational function in which work and adaptive skills are examined can be more useful than dichotomous categories.

Lastly, subjective experiences with cognitive deficits are shown to be associated with diminished occupational functioning may be an important treatment target. These experiences may point towards (sub)depressive symptoms, but also to cognitive deficits too subtle to be measured with objective instruments. This relates to the notion that the context in which subjects are objectively tested is usually more structured than everyday life. The challenges patients with bipolar disorder encounter in daily activities are more complex than the test situation. This increases the importance of cognitive abilities such as self-regulation.

### Strengths and limitations

We used a rigorous and transparent method throughout the process, which was based on the methodology as described by Arksey and O'Malley ( 2005) and further enhanced by Levac et al. (2010). Considering the iterative character of the methodology, the scoping review protocol was updated during the process. The included literature was found using an elaborate search of the literature, which ensured a broad scope on the research question. The methodology of scoping reviews allowed for the inclusion of secondary literature (e.g., systematic reviews), as well as original research, including qualitative research. As such, we could build upon the synthesis already conducted in secondary literature. Furthermore, the results from the review process stem from a multidisciplinary viewpoint including experts on occupational therapy, self-regulation, neuropsychology, bipolar disorders, and vocational rehabilitation.

The main limitation is that, following the scoping review methodology, the quality of the included literature was not assessed. Therefore, we cannot draw conclusions about the methodological rigor of included studies. Furthermore, the inclusion of secondary literature (e.g., reviews, meta-analysis) rendered a broad perspective on the conducted research, but must be interpreted with care. Most of the original research included in this scoping review is also included in the secondary literature.

## Conclusion

To conclude, many studies have found an association between EF—amongst other neurocognitive domains—in relation to occupational functioning in patients with BD. We found that there is a certain lack of conceptual clarity of both EF and occupational functioning. Still, impairments of EF are clearly a major factor in the occupational difficulties experiences in patients with BD. In most studies this relation was of a small to medium effect. However, some longitudinal studies showed a stronger association between occupational functioning and EF, especially regarding working memory, set-shifting and interference/inhibition. Considering the diminished inter-episodic functioning in many BD patients and the influence of EF impairments, enhancing neurocognitive functioning is an interesting target for clinical practice. As such, it is an important and promising area for research and treatment. Within intervention the design, the focus should shift more towards aspects of work functioning and not only work participation also taking account of the different subgroups of cognitive impairments and stages of the disorder.

## Supplementary Information

**Additional file 1: Appendix S1.** Title and abstract relevance screening tool.

**Additional file 2: Appendix S2.** Full paper relevance screening tool.

**Additional file 3: Appendix S3.** Data charting form.

**Additional file 4: Appendix S4.** Data charting form | review version.

**Additional file 5: Appendix S5.** Table complete results.

## Data Availability

The datasets used and/or analyzed during the current study are available from the corresponding author on reasonable request.

## Abbreviations

BD: Bipolar disorder
EF: Executive functions
PRISMA-ScR: PRISMA extension for scoping reviews
GAF: Global Assessment of Functioning
FAST: Functioning Assessment Short Test
