# Supplementary material for: The relations between executive functions and occupational functioning in individuals with bipolar disorder: a scoping review
Source: Int J Bipolar Disord. 2022 Mar 14;10:8. doi: 10.1186/s40345-022-00255-7 (PMC8921376; doi:10.1186/s40345-022-00255-7)
Supplement: Supplementary file 1 — Additional file 1: Appendix S1. Title and abstract relevance screening tool. [file 40345_2022_255_MOESM1_ESM.pdf]

Title and abstract relevance screening tool

Date: 30 December 2021

Search strategy

Before conducting a database search, a scope of inquiry was determined. The scope of inquiry consisted of three elements: a) population, b) concepts to be examined, c) health outcome. In this study *population* consists of individuals with bipolar disorder, *concepts* are roughly defined as executive functions and self-regulation, and *health outcome* is defined as occupational functioning. The database search strategy composed combinations of keywords relating to these three elements of the scope of inquiry, e.g. "bipolar disorder" AND "executive function*" AND "employ*".

Inclusion and exclusion criteria

In addition to article information (authors, year of publication, title, journal, and abstract) the Excel spreadsheet contains columns where reviewers can note whether an article has met the inclusion criteria:

Inclusion	*Exclusion*
Bipolar disorder (type I and II)[1];<br>Adult working population;<br>Executive functioning and/or self-regulation is researched in the context of employment;<br>Qualitative and quantitative research;<br>English language;<br>Full papers;<br>Peer reviewed.	Medication research

After reviewing title and abstract

After reviewing the title and abstract, reviewers can note whether the article matches the inclusion criteria with the following codes within the corresponding columns in the Excel spreadsheet:

0 no
1 yes
2 can't tell

This last option can be filled out when, for example, the abstract and title are not conclusive about the age of the population and the article might be relevant. When a reviewer has filled out "can't tell" the full article will be obtained for further examination. When a reviewer has filled out "no" for one of the inclusion criteria, the reviewer can stop reviewing the title and abstract and continue with the next. Additionally, the Excel spreadsheet has a column where the reviewer can note any remarks or questions relating to the title and abstract.

[1] When bipolar disorder type I or II are included in a larger sample, i.e. mood disorders, the article is still eligible for inclusion.
