# Supplementary material for: The relations between executive functions and occupational functioning in individuals with bipolar disorder: a scoping review
Source: Int J Bipolar Disord. 2022 Mar 14;10:8. doi: 10.1186/s40345-022-00255-7 (PMC8921376; doi:10.1186/s40345-022-00255-7)
Supplement: Supplementary file 2 — Additional file 2: Appendix S2. Full paper relevance screening tool. [file 40345_2022_255_MOESM2_ESM.pdf]

Full paper relevance screening tool

Date: 30 December 2021

Inclusion and exclusion criteria

In addition to article information (authors, year of publication, title, journal, and abstract) the Excel spreadsheet contains columns where reviewers can note whether an article has met the inclusion criteria:

Inclusion criteria	*Definition*
Bipolar disorder	Full-spectrum. Must be a disorder, no "manic-like experiences" without classification as bipolar disorder.
Adult working population	18-65 years.
Executive function and/or self-regulation is researched in the context of employment	Employment includes: paid employment, sheltered employment, volunteer work.
Full papers	Including comments and errata.
Peer reviewed scientific papers and dissertations	Peer review: the paper is published in a journal which uses a peer-review process where experts in the same field check the overall quality of the submitted research (e.g. design), evaluate conclusions, and identify scientific errors to ensure and improve the quality of the submitted manuscript

References

Kelly, J., Sadeghieh, T., & Adeli, K. (2014). Peer review in scientific publications: benefits, critiques, & a survival guide. *EJIFCC*, *25*(3), 227.

Smith, R. (2006). Peer review: a flawed process at the heart of science and journals. *Journal of the royal society of medicine*, *99*(4), 178-182.
