# Supplementary material for: The relations between executive functions and occupational functioning in individuals with bipolar disorder: a scoping review
Source: Int J Bipolar Disord. 2022 Mar 14;10:8. doi: 10.1186/s40345-022-00255-7 (PMC8921376; doi:10.1186/s40345-022-00255-7)
Supplement: Supplementary file 3 — Additional file 3: Appendix S3. Data charting form. [file 40345_2022_255_MOESM3_ESM.pdf]

Data charting form

Date:

Filled out by:

Reference

A. General study characteristics		
Variable	*Category*	*Explanation*
1. Publication type	Publication type:<br>☐ Journal article<br>☐ Thesis dissertation<br>☐ Secondary research (specify type*: _______________)	* E.g. systematic review, meta-analysis, narrative review, scoping review.
2. Study sector setting	☐ Psychiatry<br>☐ Neuropsychology<br>☐ Rehabilitation<br>☐ Other, please specify:<br>______________________	Check all that apply
3. Study design	☐ Longitudinal, please specify:<br>☐ Cross-sectional<br>☐ RCT<br>☐ Intervention research, please specify which intervention is examined:<br>______________________<br>☐ Other, please specify:<br>______________________	Check all that apply
4. Objective		Please copy paste the aim of the study as stated by the authors.
5. Country of origin		Please specify the country in which the study was conducted.

B. Study population		
Variable	*Category*	*Explanation*
6. Diagnostic groups	☐ BD I	Check all that apply.

	☐ BD II<br>☐ BD NAO<br>☐ BD, type not specified<br>☐ Mixed group, please specify*:<br>________________________	* E.g. broad group of SMI, not separately reported for bipolar disorder
7. Number of participants	N=	Please specify the N of each diagnostic (bipolar) group.
8. Healthy control group	Is there a healthy control group?<br>☐ Yes, please specify number of healthy controls:<br>________________________<br>☐ No	
9. Diagnosis check	☐ SCID<br>☐ Clinical diagnosis<br>☐ Not specified / not reported<br>☐ Other, please specify:<br>________________________	Check all that apply.
10. Age	Is the age range of the study population reported?<br>☐ Yes, please specify:<br>________________________<br>☐ No	
11. Gender	Was the ratio man-woman reported?<br>☐ Yes, please specify:<br>________________________<br>☐ No	
12. Episodes	Is the current mood state of participants reported?<br>☐ Depression<br>☐ Hypomania<br>☐ Mania<br>☐ Euthymia<br>☐ Not specified<br>☐ Other, please specify:<br>________________________	Please specify the current mood state of participants as stated by the author(s).<br>Check all that apply.

C. Executive functioning and self-regulation		
Variable	*Category*	*Explanation*

13. Definition	Is executive functioning and/or self-regulation defined in the study?<br>☐ Yes, please specify:<br>______________________<br>☐ No	Please copy-paste the definition as stated in the report.
14. Measurements	☐ Tower of London<br>☐ Trail Making Test-A (TMT-A)<br>☐ Trail Making Test-B (TMT-B)<br>☐ Stroop Color and Word Test<br>☐ Wisconsin Card Sorting Test (WCST)<br>☐ Other, please specify:<br>______________________	Check all that apply.
15. Choice for measurements	☐ Not reported<br>☐ Reported, please specify:<br>______________________	Please specify the reasoning of the authors for the employed measurements.
16. Results	Which results on executive functions are reported?	Please describe which results (e.g. impairments or comparison to healthy controls) have been reported in individuals with bipolar disorder in the study.
17. Degree of impairments	☐ Not reported<br>☐ Reported, please specify*:<br>______________________	*Please describe what the author(s) describe regarding the degree of measured impairments in executive functioning.

D. Vocational functioning		
Variable	*Category*	*Explanation*
18. Definition	Is vocational functioning defined in the study?<br>☐ Yes, please specify:<br>☐ No	Please copy-paste the definition as stated in the report.
19. Measurements	☐ SOFAS<br>☐ Life Functioning Questionnaire (LFQ)<br>☐ UCSD Performance-Based Skills Assessment<br>☐ Health Performance Questionnaire (HPQ)<br>☐ WHODAS 2.0<br>☐ WHOQoL	Check all that apply.

	☐ Modified Vocational Index<br>☐ Other, please specify:<br>________________________	
20. Level of vocational functioning	☐ Sheltered employment<br>☐ Volunteer work<br>☐ Paid employment<br>☐ Unemployed<br>☐ Not reported<br>☐ Other, please specify:<br>________________________	
21. Results	Which results regarding vocational functioning are reported?	Please specify what the authors have reported regarding results of measurements on vocational functioning. This depends on how the authors have measured vocational functioning, e.g. only level of employment as outcome as opposed to a more detailed measurement.
22. Degree of impairments	☐ Not reported<br>☐ Reported, please specify*:<br>________________________	*Please describe what the author(s) describe regarding the degree of measured impairments in vocational functioning.
23. Context	☐ Context of employment has been described, please specify:<br>________________________<br>☐ Context has not been described	Please describe whether or not the authors have reported about the context of employment. E.g. characteristics of the work environment, which employment sector.

E. Relationships		
Variable	*Category*	*Explanation*
24. Qualitative analysis	What kind of qualitative methodology is used?<br>☐ Narrative<br>☐ Grounded theory<br>☐ Phenomological<br>☐ Qualitative description*<br>☐ Not specified<br>☐ No qualitative data used<br>☐ Other, please specify:<br>________________________	Check all that apply.<br>* There is no specific methodology mentioned, but there is a qualitative data collection and analysis.
25. Statistical analysis	What kind of statistical analysis is reported?	Check all that apply.

	☐ Descriptive statistics<br>☐ Inferential univariate analysis<br>☐ Inferential multivariate analysis<br>☐ No quantitative data used	
26. Strength of the relationship (qualitative and/or quantitative)	What is reported about the strength of the relationship between variables?	For qualitative data: please copy paste what the authors reported about the substantiation of their hypothesis.
27. Results		Please describe (or copy-paste) the results of examining the relationship between the examined variables.

F. Additional information	
28. Please describe any additional relevant information here.	-
