# Supplementary material for: The relations between executive functions and occupational functioning in individuals with bipolar disorder: a scoping review
Source: Int J Bipolar Disord. 2022 Mar 14;10:8. doi: 10.1186/s40345-022-00255-7 (PMC8921376; doi:10.1186/s40345-022-00255-7)
Supplement: Supplementary file 4 — Additional file 4: Appendix S4. Data charting form | review version. [file 40345_2022_255_MOESM4_ESM.pdf]

Data charting form | review version

Date:

Filled out by:

Reference

A. General study characteristics		
Variable	*Category*	*Explanation*
1. Publication type	Publication type:<br>☐ Systematic review<br>☐ Meta-analysis<br>☐ Qualitative review<br>☐ Other*, please specify:<br>_______________	* E.g. narrative review, scoping review.
2. Study sector setting	☐ Psychiatry<br>☐ Neuropsychology<br>☐ Rehabilitation<br>☐ Other, please specify:<br>______________________	Check all that apply
3. Review design / method	☐ Guideline or framework is used, please specify:<br>______________________<br>☐ Not reported	Check all that apply
4. Number of included papers	☐ Number:<br>☐ Not reported	Please specify the number of included papers as stated by the authors.
5. Time frame	☐ Time frame:<br>☐ Not reported	Please specify the reported time frame of the search.
6. Objective		Please copy paste the aims of the study as stated by the authors.
7. Country of origin		Please specify the country in which the review was conducted.

B. Study population		
Variable	*Category*	*Explanation*
8. Diagnostic groups	☐ BD I<br>☐ BD II	Check all that apply.

	☐ BD NAO<br>☐ BD, type not specified<br>☐ Mixed group, please specify*:<br>________________________	* E.g. broad group of SMI, not separately reported for bipolar disorder
9. Number of participants	☐ N=<br>☐ Not applicable<br>☐ Not reported	Please specify the N of each diagnostic (bipolar) group.
10. Age	Is the age range of the study population reported?<br>☐ Yes, please specify:<br>________________________<br>☐ No	
11. Gender	Was the ratio man-woman reported?<br>☐ Yes, please specify:<br>________________________<br>☐ No	

C. Executive functioning and self-regulation		
Variable	*Category*	*Explanation*
12. Definition	Is executive functioning and/or self-regulation defined in the study?<br>☐ Yes, please specify:<br>________________________<br>☐ No	Please copy-paste the definition as stated in the report.
13. Measurements	☐ Tower of London<br>☐ Trail Making Test-A (TMT-A)<br>☐ Trail Making Test-B (TMT-B)<br>☐ Stroop Color and Word Test<br>☐ Wisconsin Card Sorting Test (WCST)<br>☐ Other, please specify:<br>________________________	Check all that apply.
14. Results / conclusions	Which results on executive functions are reported?	Please describe the most important results and conclusions of the review / meta-analysis.

D. Vocational functioning		
Variable	*Category*	*Explanation*

15. Definition	Is vocational functioning defined in the study?<br>☐ Yes, please specify:<br>☐ No	Please copy-paste the definition as stated in the report.
16. Measurements	☐ SOFAS<br>☐ Life Functioning Questionnaire (LFQ)<br>☐ UCSD Performance-Based Skills Assessment<br>☐ Health Performance Questionnaire (HPQ)<br>☐ WHODAS 2.0<br>☐ WHOQoL<br>☐ Modified Vocational Index<br>☐ Other, please specify:<br>________________________	Check all that apply.
17. Level of vocational functioning	☐ Sheltered employment<br>☐ Volunteer work<br>☐ Paid employment<br>☐ Unemployed<br>☐ Not reported<br>☐ Other, please specify:<br>________________________	
18. Results / conclusions	Which results regarding vocational functioning are reported?	Please describe the most important results and conclusions of the review / meta-analysis.
19. Context	☐ Context of employment has been described, please specify:<br>________________________<br>☐ Context has not been described	Please describe whether or not the authors have reported about the context of employment. E.g. characteristics of the work environment, which employment sector.

E. Relationships		
Variable	*Category*	*Explanation*
20. Qualitative analysis	What kind of qualitative methodology is used?<br>☐ Narrative<br>☐ Grounded theory<br>☐ Phenomenological<br>☐ Qualitative description*	Check all that apply.<br>* There is no specific methodology mentioned, but there is a qualitative data collection and analysis.

	☐ Not specified<br>☐ No qualitative data used<br>☐ Other, please specify:<br>________________________	
21. Statistical analysis	What kind of statistical analysis is reported?<br>☐ Descriptive statistics<br>☐ Inferential univariate analysis<br>☐ Inferential multivariate analysis<br>☐ No quantitative data used<br>☐ Other, please specify:<br>________________________	Check all that apply.
22. Strength of the relationship (qualitative and/or quantitative)	What is reported about the strength of the relationship between variables?	For qualitative data: please copy paste what the authors reported about the substantiation of their hypothesis.
23. Results		Please describe (or copy-paste) the results of examining the relationship between the examined variables.

F. Additional information	
24. Please describe any additional relevant information here.	-
